# Childhood Maltreatment and Psychosocial Flourishing among Emerging Adults: Roles of Psychological *Suzhi* and Self-Esteem

**DOI:** 10.3390/ijerph19094998

**Published:** 2022-04-20

**Authors:** Zewei Li, Yangu Pan, Guangzeng Liu, Bingbing Li, Xu Li

**Affiliations:** 1 Department of Psychology, Educational College, Shanghai Normal University, Shanghai 200234, China; zeweil_0410@163.com (Z.L.); libb86@shnu.edu.cn (B.L.); 2 Research Institute of Social Development, Southwestern University of Finance and Economics, Chengdu 611130, China; panyg@swufe.edu.cn; 3 School of Management, Chongqing University of Technology, Chongqing 400054, China; lgzohyeah@163.com

**Keywords:** childhood maltreatment, psychological *suzhi*, self-esteem, psychosocial flourishing

## Abstract

Studies have shown that childhood maltreatment can negatively predict psychosocial flourishing among emerging adults. However, few studies have revealed the factors that can protect the psychosocial flourishing of emerging adults who experienced maltreatment during childhood. Based on theoretical and empirical considerations, this study investigated whether and how psychological *suzhi* (a positive quality that can facilitate individuals’ adaptation to environment) plays a protective role in the relationship between childhood maltreatment and psychosocial flourishing among emerging adults. A total of 2863 Chinese emerging adults (*M*_age_ = 19.96 years) completed the self-report measures of the Childhood Trauma Questionnaire, Psychological *Suzhi* Questionnaire, Rosenberg Self-esteem Scale, and Flourishing Scale. The moderated analyses showed that childhood maltreatment had a less negative impact on psychological flourishing in high psychological *suzhi* emerging adults than in low psychological *suzhi* emerging adults. Part of the moderating effect of psychological *suzhi* is mediated through self-esteem. These results demonstrated that psychological *suzhi* plays a buffering effect between childhood maltreatment and psychosocial flourishing, and part of the effect is achieved by mediated variable self-esteem. These findings are discussed, and practical implications are presented.

## 1. Introduction

Childhood maltreatment refers to individuals’ adverse experiences during childhood, including physical, sexual, and emotional abuse and neglect [1]. Recent studies have shown that childhood maltreatment can significantly reduce the well-being of emerging adults (from 18 to 29-years old) [2], such as subjective well-being [3], psychological well-being [4], social well-being [5], and psychosocial flourishing [6]. Psychosocial flourishing is a more comprehensive indicator of well-being. Flourishing individuals show self-acceptance, positive interpersonal relationships, competence, meaning, purpose in life and contribute to the well-being of others [7]. These psychosocial resources will help emerging adults solve many challenges they face in life, such as academic pressure, establishing intimate relationships, maintaining good interpersonal relationships, and employment pressure [8]. Therefore, it is necessary to reveal the factors that play a protective role in the relationship between childhood maltreatment and psychosocial flourishing among emerging adults, which previous studies have not addressed. Answering this question will have significant implications for alleviating the negative impact of childhood maltreatment on psychosocial flourishing.

### 1.1. Psychological Suzhi as a Moderator

Differential susceptibility theory states that some people are more susceptible to positive and negative environmental conditions than others [9]. In adverse environments (e.g., childhood maltreatment), individuals’ positive characteristics are considered protective factors that can buffer the negative effect of adversity on development [10,11]. Therefore, we assumed that the effect of childhood maltreatment on psychosocial flourishing may be contingent upon the positive characteristics of individuals such as psychological *suzhi*.

Psychological *suzhi* is based on physiological conditions; it involves the internalization of externally obtained stimuli as stable, basic, and derivative psychological qualities, which are closely related to individuals’ adaptation and development [12]. Psychological *suzhi* includes three dimensions: cognitive quality, individuality, and adaptability [13]. Cognitive qualities refer to the qualities that an individual exhibits in cognitive activities, including thinking qualities (e.g., agility, fluency, and flexibility) and metacognition. Individuality comprises the psychological characteristics that an individual exhibits in practical activities, including conscientiousness, optimism, and volition (e.g., independence and persistence). Adaptability is the notion of emotional and interpersonal skills and is flexible in different social contexts, so it governs individuals’ ability to achieve harmony between themselves and the environment. Psychological *suzhi* is a concept that combines content elements (e.g., cognitive quality and personality) and functional elements (e.g., adaptability). Taken together, psychological *suzhi* reflects the positive psychological traits that facilitate students’ positive adaptation to the school and social environment [14].

Zhang and Wang [15] proposed that psychological *suzhi* can buffer the negative effects of early adverse experiences on individuals’ adaptation and development. Research has found that psychological *suzhi* moderates the impact of bullying victimization on children’s social anxiety, that is, compared with children with high psychological *suzhi*, low psychological *suzhi* children show higher social anxiety after being bullied and victimized [16]. In another study, the negative impact of using multiple media tools on sleep quality among adolescents with low psychological *suzhi* was significantly greater than its impact on adolescents with high psychological *suzhi* [17]. Based on previous findings, this study hypothesizes that psychological *suzhi* may play a moderating role between childhood maltreatment and psychosocial flourishing, that is, childhood maltreatment, has a less negative impact on the psychosocial flourishing of emerging adults with high psychological *suzhi* than adults with low psychological *suzhi*.

### 1.2. Self-Esteem as a Mediator

If the predictive effect of childhood maltreatment on psychosocial flourishing is moderated by psychological *suzhi*, the next question that needs to be answered is related to how psychological *suzhi* works: what are the underlying mechanisms of the moderating effect of psychological *suzhi*? To answer this question, a mediated moderation model is proposed in this study. In this model, at least a partial moderating effect is explained by a mediator variable.

If a variable plays a mediating role in the moderating effect of psychological *suzhi*, two aspects of the evidence are required [18]. First, psychological *suzhi* can moderate the association between childhood maltreatment and this variable. Second, this variable can significantly predict psychosocial flourishing. If a variable can meet the above conditions, then it is the variable that mediates the moderating effect of psychological *suzhi*. According to ecological theory [19], compared with environmental variables such as childhood maltreatment, individual variables may be proximal variables affecting psychosocial flourishing. Therefore, we supposed that an individual variable might be a mediated variable.

Self-esteem, defined as a general sense of one’s worth, is an important individual variable [20]. Individuals with high self-esteem have a positive attitude towards themselves and believe that they are capable and respected [20]. Self-determination theory states that competence and relatedness are basic psychological needs essential for well-being [21]. In addition, self-acceptance is an important manifestation of psychosocial flourishing [7]. Based on these theoretical considerations, high self-esteem should be a prerequisite for psychosocial flourishing. Indeed, previous studies have found that self-esteem can significantly positively predict psychosocial flourishing among adolescents [22] and emerging adults [6,23]. A longitudinal study also found that self-esteem could promote psychosocial flourishing [24].

Evidence is also needed to support the moderating role of psychological *suzhi* in the relationship between childhood maltreatment and self-esteem. As a positive indicator of personal development, self-esteem is the core of mental health [25,26]. The negative impact of adverse environments on individual adaptation and development can be buffered by psychological *suzhi* [15]. Therefore, psychological *suzhi* should protect the development of self-esteem when an individual experiences childhood maltreatment. Taken together, we hypothesized that the moderating effect of psychological *suzhi* in the association between childhood maltreatment and psychosocial flourishing may be achieved through mediated variable self-esteem. Specifically, we assumed that the self-esteem of individuals with high psychological *suzhi* was less damaged by childhood maltreatment than those with low psychological *suzhi*, and self-esteem could significantly positively predict psychosocial flourishing.

### 1.3. The Hypotheses of the Study

**Figure 1:**
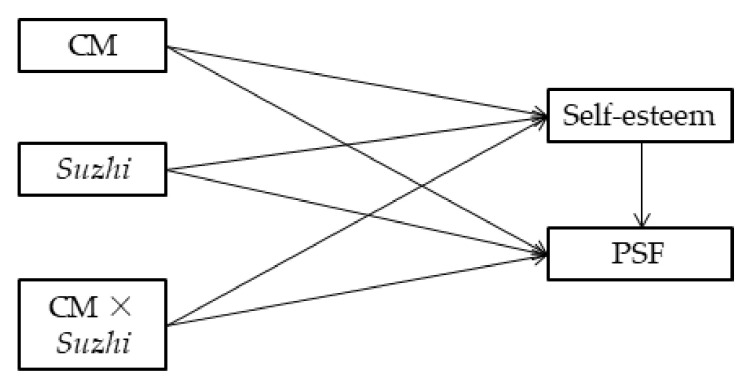
The mediated moderation model of the influence of childhood maltreatment on psychosocial flourishing. Note: CM = childhood maltreatment; *Suzhi* = psychological suzhi; PSF = psychosocial flourishing.

In summary, this study intends to reveal the factor that can buffer the negative effect of childhood maltreatment on psychosocial flourishing among emerging adults, and the underlying mechanism of the buffering effect. The first hypothesis is that psychological *suzhi* can moderate the negative effect of childhood maltreatment on psychosocial flourishing; that is, childhood maltreatment has a less negative impact on the psychosocial flourishing of emerging adults with high psychological *suzhi* than adults with low psychological *suzhi*. The second hypothesis is that self-esteem mediates part of the moderating effect of psychological *suzhi* (Figure 1).

## 2. Method

### 2.1. Participants and Procedure

This study conducted a questionnaire survey on 2992 undergraduate students from 14 universities in eight provinces of China. A total of 2863 participants were included in data analyses (*M*_age_ = 19.96 years, *SD* = 1.78 years; 1716 female and 1147 male). Data for 129 participants were excluded due to missing or extreme values. These participants are 4.3% of the sample, and it will have little influence on the result obtained [27]. These number of freshmen, sophomores, juniors, and seniors were 1073 (35.9%), 703 (23.5%), 548 (18.3%) and 539 (18.0%), respectively.

Participants completed a set of questionnaires by the Questionnaire Star program in quiet classrooms. The researcher explained the instructions for filling out the questionnaires to participants, and participants’ names do not need to be filled in the measures to ensure data quality. All questionnaires can be completed in about 10 min. Each participant will get cash compensation after the survey is over. This study was approved by the institutional review board of Shanghai Normal university. The procedures used in this study adhere to the tenets of the Declaration of Helsinki. All participants provided oral consent.

### 2.2. Measures

#### 2.2.1. Childhood Maltreatment

Childhood maltreatment was measured by the Childhood Trauma Questionnaire-Short Form (CTQ-SF) [1]. The CTQ-SF is a widely used instrument that measures maltreatment in childhood. The CTQ-SF contains the subscales of emotional abuse, physical abuse, and sexual abuse, emotional neglect, and physical neglect. Each subscale was assessed by five items scored on a 5-point Likert scale ranging from 1 (never) to 5 (very often). A sample item on physical abuse is as follows: “My parents hit me enough to see doctor”. To measure the prevalence of each type of childhood maltreatment, the criteria used in this study were: emotional abuse ≥ 9, emotional neglect ≥ 10, physical abuse ≥ 8, physical neglect ≥ 8, and sexual abuse ≥ 6. This criterion was also used in Chinese samples by several studies [28,29,30]. This study used the validated Chinese vision of CTQ-SF [31]. In the present study, the Cronbach’s alpha coefficient was 0.73 for the whole scale.

#### 2.2.2. Psychological Suzhi

Psychological *Suzhi* was measured by the simplified version of the Psychological *Suzhi* Questionnaire for Undergraduate Students (PSQUS) [13]. The PSQUS contains 27 items examining three dimensions: cognitive quality (e.g., “I can think of a variety of uses for an item”), individuality (e.g., “I always feel that life is full of fun”), and adaptability (e.g., “I have clear goals and know what I should do every day”). Each dimension was assessed by nine items scored on a 5-point Likert scale ranging from 1 (totally disagree) to 5 (totally agree). In the present study, the Cronbach’s alpha coefficient for the whole scale was 0.94.

#### 2.2.3. Self-Esteem

Self-esteem was measured by the Rosenberg Self-esteem Scale (RSES) [20]. All 10 items were scored on a 4-point Likert scale (1 = strongly disagree; 4 = strongly agree). Sample item from RSES is “I can do things well like most people”. In this study, the Cronbach’s alpha coefficient was 0.91.

#### 2.2.4. Psychosocial Flourishing

Psychosocial flourishing was measured by the Flourishing Scale [7]. This scale consists of eight items describing important aspects of human positive functioning as follows: positive interpersonal relationships, self-acceptance, competence, meaning and purpose in life, and contributing to the well-being of others. A Sample item from the Flourishing Scale is “I am competent in the activities that are important to me”. Each item is answered on a 7-point Likert scale (1 = strongly disagree; 7 = strongly agree). In the present study, the Cronbach’s alpha coefficient was 0.94.

### 2.3. Data Analysis

The skewness and kurtosis of psychosocial flourishing, childhood maltreatment, psychological *suzhi*, and self-esteem fell within the acceptable range (skewness < 2.0 and kurtosis < 7.0) [32]. What’s more, the bootstrapping method was used to calculate estimators, so the normality of data distribution has little effect on it [33]. The Statistical Package for Social Sciences (SPSS) 21.0 software was used to conduct common method deviation test, descriptive statistics and correlation analysis on the data. Gender and grade differences in psychosocial flourishing were analyzed using an independent samples *t*-test and analysis of variance (ANOVA), respectively. Following the method of model analysis [18], SPSS PROCESS plug [34] and Bootstrap test method (5000 sampling with replacement) were adopted to estimate the parameters of three regression equations to test whether the mediated moderation holds. In each equation, all the predictors were centralized to reduce any multicollinearity [35].

## 3. Results

### 3.1. Common Method Deviation Test

The Harman’s single factor test was used to test for common method deviation. The result showed that the eigenvalues of 14 factors were greater than one. The explanatory variation of the most important factor was 22.5%, which is less than the critical standard of 40% [36]. Therefore, there is no obvious common method deviation in the present study.

### 3.2. Preliminary Analysis

The results of descriptive statistics and Pearson’s bivariate correlation for all variables are shown in Table 1. 

In the sample of this study, the proportion of participants with an average CTQ-SF score greater than 1 (that is, subjects who experienced abuse or neglect) was 82.4%. The proportion of participants who reached the critical standard was 48.2%. The detection rate was 6% for physical abuse, 15% for emotional abuse, 27.5% for physical neglect, 21.9% for emotional neglect, and 15.4% for sexual abuse.

The ANOVA found grade effects on psychosocial flourishing, childhood maltreatment, psychological *suzhi*, and self-esteem, while all effect sizes are small (partial η^2^ < 0.01). Therefore, these grade effects have no practical significance. Gender differences in self-esteem, psychological *suzhi*, and childhood maltreatment were tested to be significant, but all effect sizes are small (d < 0.2). Thus, these gender effects have no practical significance. In addition, there was no significant difference in psychosocial flourishing between boys and girls, *t* (2861) = 1.55, *p* = 0.12, *d* = 0.06. Therefore, grade and gender were not included as control variables in subsequent model tests.

### 3.3. Testing for Mediated Moderation

**Figure 2:**
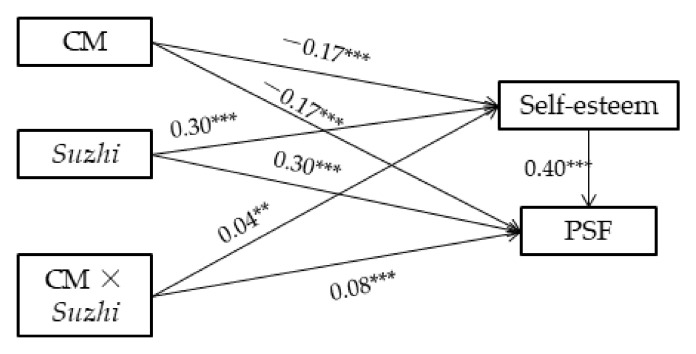
The regression coefficients of the mediated moderation model of the influence of childhood maltreatment on psychosocial flourishing. Note: CM = childhood maltreatment; *Suzhi* = psychological *suzhi*; PSF = psychosocial flourishing. ** *p* < 0.01. *** *p* < 0.001.

The parameters for three regression equations were estimated to test the mediated moderation hypothesis [18]. Regression Equation (1) was conducted by the model 1 of SPSS PROCESS plugin [34], and the regression coefficients of psychosocial flourishing on childhood maltreatment, psychological *suzhi*, and their interaction were estimated. Regression Equations (2) and (3) were conducted by the model 8 of SPSS PROCESS plugin [34]. The regression coefficients of self-esteem on childhood maltreatment, psychological *suzhi*, and their interaction were estimated in Equation (2). The regression coefficients of psychosocial flourishing on childhood maltreatment, psychological *suzhi*, and their interaction and self-esteem were estimated in Equation (3). The regression coefficients of the three regression equations and mediated moderation model are presented in Table 2 and Figure 2, respectively. If both of the regression coefficients of the interaction of childhood maltreatment and psychological *suzhi* in Equations (1) and (2) are significant, and the predictive effect of self-esteem on psychosocial flourishing in Equation (3) is significant, then there is a mediated moderating effect [18].

As shown in Table 2, in Equations (1) and (2), the interaction between childhood maltreatment and psychological *suzhi* had significant predictive effects on psychosocial flourishing (*β* = 0.09, *SE* = 0.013, *p* < 0.001, 95% CI [0.068, 0.117]) and self-esteem (*β* = 0.04, *SE* = 0.014, *p* < 0.01, 95% CI [0.017, 0.072]), respectively. In Equation (3), the effect of self-esteem on psychosocial flourishing was significant (*β* = 0.40, *SE* = 0.015, *p* < 0.001, 95% CI [0.369, 0.428]), and the effect of the interaction of childhood maltreatment and psychological *suzhi* on psychosocial flourishing was significant (*β* = 0.08, *SE* = 0.011, *p* < 0.001, 95% CI [0.053, 0.097]). These findings indicate that the mediated moderation model is established, and part of the moderating effect between childhood maltreatment and psychological *suzhi* was mediated by self-esteem.

**Figure 3:**
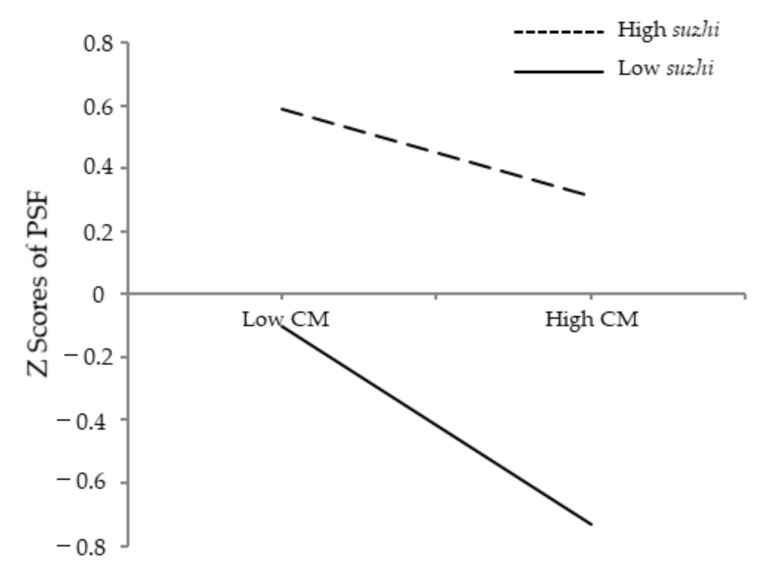
Psychological *suzhi* as a moderator of the relationship between childhood maltreatment and psychosocial flourishing. Note: CM = childhood maltreatment; *suzhi* = psychological *suzhi*; PSF = psychosocial flourishing.

Figure 3 presents the interaction effect of childhood maltreatment and psychological *suzhi* on psychosocial flourishing for descriptive purposes. Simple slope analysis found that the negative effect of childhood maltreatment on psychosocial flourishing was significant for low psychological *suzhi* participants (1 *SD* below the mean), *β* = −0.32, *SE* = 0.018, 95% CI = [−0.351, −0.281], *p* < 0.001, while the strength of the effect became weak for high psychological *suzhi* participants (1 *SD* above the mean), *β* = −0.14, *SE* = 0.023, 95% CI = [−0.193, −0.107], *p* < 0.001.

**Figure 4:**
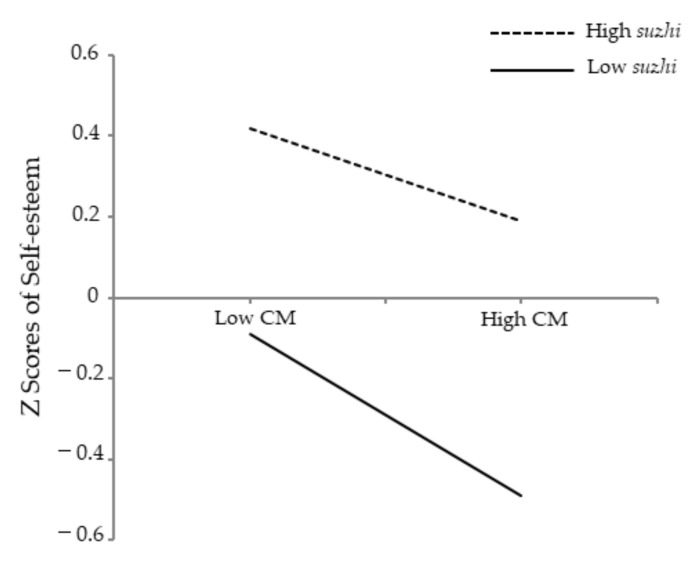
Psychological *suzhi* as a moderator of the relationship between childhood maltreatment and self-esteem. Note: CM = childhood maltreatment; *suzhi* = psychological *suzhi*.

Figure 4 presents the interaction effect of childhood maltreatment and psychological *suzhi* on self-esteem. Simple slope analysis found that the negative effect of childhood maltreatment on self-esteem was significant for low psychological *suzhi* participants (1 *SD* below the mean), *β* = −0.20, *SE* = 0.020, 95% CI = [−0.243, −0.166], *p* < 0.001, while the strength of the effect became weak for high psychological *suzhi* participants (1 *SD* above the mean), *β* = −0.13, *SE* = 0.025, 95% CI = [−0.174, −0.077], *p* < 0.001.

## 4. Discussion

This study aimed to reveal the factors that play a protective role in the relationship between childhood maltreatment and psychosocial flourishing among emerging adults and to examine its potential mechanisms. Based on the theory of differential susceptibility, this study examined a mediated moderation model. In this model, we assume that psychological *suzhi* moderates the negative effect of childhood maltreatment on psychosocial flourishing and that self-esteem mediates the moderating effect.

Consistent with Hypothesis 1, the study results found that psychological *suzhi* plays a moderating role between childhood maltreatment and psychosocial flourishing. Specifically, compared with the high psychological *suzhi* group, the negative predictive effect of childhood maltreatment on psychosocial flourishing was stronger in the low psychological *suzhi* group. This result reveals that psychological *suzhi* can moderate the strength of the association between childhood maltreatment and psychosocial flourishing, expanding the existing research on the relationship between childhood maltreatment and well-being [3,5,6]. This finding enriches the differential susceptibility theory by revealing important individual factors (e.g., psychological *suzhi*) that interact with environmental factors (e.g., childhood maltreatment) in developing psychosocial flourishing among emerging adults. Consistent with our findings, previous studies have found a moderating effect of psychological *suzhi*. Specifically, the negative effect of school climate on alcohol use among low psychologically *suzhi* adolescents was significantly greater than its effect on high psychologically *suzhi* adolescents [17]. Compared with children with low psychological *suzhi*, bullying victimization has less impact on social anxiety in children with high psychological *suzhi* [16]. Taken together, these results suggest that psychological *suzhi* can weaken the risk effect of childhood maltreatment on psychosocial flourishing among emerging adults.

This research further reveals the potential mechanisms of the moderating effect of psychological *suzhi*. Consistent with Hypothesis 2, the results showed that psychological *suzhi* significantly moderated the relationship between childhood maltreatment and self-esteem, and self-esteem had a significant positive predictive effect on psychosocial flourishing. Specifically, childhood maltreatment had a less negative impact on self-esteem in high psychological *suzhi* emerging adults than in low psychological *suzhi* emerging adults. These findings demonstrate that self-esteem mediates the moderating effect of psychological *suzhi* on the association between childhood maltreatment and psychosocial flourishing. These findings also expand previous research that did not address how psychological *suzhi* protects against the risk of consequences from adverse environmental factors [16,37]. These findings are probably because high psychological *suzhi* emerging adults use more adaptive cognition and coping strategies (e.g., positive emotion regulation strategies) [38] when they experience maltreatment. Therefore, they can maintain a good state and have a positive self-worth evaluation, enhancing psychosocial flourishing. Indeed, studies have shown that middle school students with high psychological *suzhi* use more cognitive reappraisal emotion regulation strategies [39] and show higher levels of self-esteem [40]. In addition, the results found that the interaction effect between childhood maltreatment and psychological *suzhi* on psychosocial flourishing was significant when self-esteem was included in Equation (3), indicating that self-esteem partly accounted for the moderating effect found between childhood maltreatment and psychological *suzhi*. Taken together, these findings suggest that psychological *suzhi* can protect self-esteem and psychosocial flourishing among maltreated emerging adults, supporting the view that psychological *suzhi* can facilitate Chinese students’ positive adaptation to the environment [12].

This study has some limitations, and recommendations are presented for future research. First, the cross-sectional design used in this study cannot infer causal relationships among the examined variables and a longitudinal study design should thus be conducted to test it. Second, all variables were measured by self-reported scales; future studies should include information from relevant others (e.g., parents, teachers, and peers) and use a wider variety of data collection methods (e.g., observations and interviews) to increase the validity of research results. Third, the sampling of this study takes undergraduate students to represent emerging adults; future research can expand the sampling scope and improve the representativeness of the sample.

Psychological *suzhi* was proposed within the context of Chinese quality-oriented education [12]. It is a positive psychological trait which overlaps with the concept of resilience proposed by Western psychologists [41]. Previous studies have found that both psychological *suzhi* and resilience can alleviate the negative impact of bullying on children’s social anxiety [16]. Therefore, the protective effects of psychological *suzhi* found in this study should also apply to emerging adults from different cultural backgrounds. The findings of this study extend our understanding of the impact of childhood maltreatment on emerging adults’ psychosocial flourishing, which has significant implications for prevention efforts. Specifically, enhancing psychological *suzhi* should focus on preventive interventions to mitigate the negative impact of childhood maltreatment on psychosocial flourishing among emerging adults. Zhang and colleagues have developed some effective training models and implementation strategies to enhance psychological *suzhi*, including specific problem-oriented training models, subject infiltration, computer-assisted training models, home-school cooperation, and aesthetic edification [42]. The results also suggest that treatments designed to cultivate self-esteem may also be promising for enhancing psychosocial flourishing. Studies have shown that mindfulness positively affects self-esteem [43,44]. Mindfulness saves individuals from negative beliefs or critical feelings that represent low self-esteem [45,46]. Therefore, meditation or mindfulness training that increases the level of mindfulness will be beneficial for individuals’ self-esteem.

## 5. Conclusions

In conclusion, this study examined the role of psychological *suzhi* and self-esteem in the relationship between childhood maltreatment and psychosocial flourishing among emerging Chinese adults. Psychological *suzhi* can buffer the negative impact of childhood maltreatment on psychosocial flourishing; that is, compared with high psychological *suzhi* emerging adults, childhood maltreatment is more likely to damage psychosocial flourishing in low psychological *suzhi* emerging adults. Self-esteem partly mediates the moderating effect between childhood maltreatment and psychological *suzhi*. These findings have important implications for prevention efforts to alleviate the negative effects of childhood maltreatment on psychosocial flourishing.

## Figures and Tables

**Table 1:** Means, standard deviations (*SD*), and correlations of all variables (*n* = 2863).

Variable	*Mean*	*SD*	1	2	3	4
1. CM	31.50	7.04	-			
2. *Suzhi*	103.06	13.35	−0.29 **	-		
3. Self-esteem	27.92	5.45	−0.27 **	0.35 **	-	
4. PSF	47.33	7.67	−0.38 **	0.50 **	0.56 **	-

Note. CM = childhood maltreatment; *Suzhi* = psychological *suzhi*; PSF = psychosocial flourishing. ** *p* < 0.01.

**Table 2:** Testing the mediated moderation effects of childhood maltreatment on psychosocial flourishing (*n* = 2863).

Predictors	Equation (1) (PSF)		Equation (2) (Self-Esteem)		Equation (3) (PSF)	
	*β*	*t*	*β*	*t*	*β*	*t*
CM	−0.23	−14.08 ***	−0.17	−8.93 ***	−0.17	−11.13 ***
*Suzhi*	0.42	26.37 ***	0.30	16.76 ***	0.30	20.14 ***
CM × *Suzhi*	0.09	7.39 ***	0.04	3.15 **	0.08	6.67 ***
Self-esteem					0.40	26.61 ***
*R* ^2^	0.33		0.16		0.46	
*F*	460.06 ***		177.11 ***		607.31 ***	

Note: PSF = psychosocial flourishing; CM = childhood maltreatment; *Suzhi* = psychological *suzhi*. Each column is a regression equation that predicts the dependent variable at the top of the column. The beta values are standardized coefficients. ** *p* < 0.01. *** *p* < 0.001.

## Data Availability

The data of the present study supporting the conclusions will be made available on request by the corresponding authors.

## References

[1] Bernstein D.P., Stein J.A., Newcomb M.D., Walker E., Pogge D., Ahluvalia T., Stokes J., Handelsman L., Medrano M., Desmond D. (2003). Development and validation of a brief screening version of the Childhood Trauma Questionnaire. Child Abus. Negl..

[2] Arnett J.J. (2007). Emerging Adulthood: What Is It, and What Is It Good For?. Child Dev. Perspect..

[3] Wu Q.L., Cao H.J., Lin X.Y., Zhou N., Chi P.L. (2021). Child Maltreatment and Subjective Well-being in Chinese Emerging Adults: A Process Model Involving Self-esteem and Self-compassion. J. Interpers. Violence.

[4] Tarber D.N., Cohn T.J., Casazza S., Hastings S.L., Steele J. (2016). The Role of Self-compassion in Psychological Well-being for Male Survivors of Childhood Maltreatment. Mindfulness.

[5] Arslan G. (2021). Psychological maltreatment predicts decreases in social wellbeing through resilience in college students: A conditional process approach of positive emotions. Curr. Psychol..

[6] Li B.B., Pan Y.G., Liu G.Z., Chen W.F., Lu J.M., Li X. (2020). Perceived social support and self-esteem mediate the relationship between childhood maltreatment and psychosocial flourishing in Chinese undergraduate students. Child Youth Serv. Rev..

[7] Diener E., Wirtz D., Tov W., Kim-Prieto C., Choi D.W., Oishi S., Biswas-Diener R. (2010). New Well-being Measures: Short Scales to Assess Flourishing and Positive and Negative Feelings. Soc. Indic. Res..

[8] Kretschmer T., Veenstra R., Branje S., Reijneveld S.A., Meeus W.H.J., Dekovic M., Koot H.M., Vollebergh W.A.M., Oldehinkel A.J. (2018). How Competent are Adolescent Bullying Perpetrators and Victims in Mastering Normative Developmental Tasks in Early Adulthood?. J. Abnorm. Child Psychol..

[9] Ellis B.J., Boyce W.T., Belsky J., Bakermans-Kranenburg M.J., van Ijzendoorn M.H. (2011). Differential susceptibility to the environment: An evolutionary-neurodevelopmental theory. Dev. Psychopathol..

[10] Masten A.S., Narayan A.J. (2012). Child Development in the Context of Disaster, War, and Terrorism: Pathways of Risk and Resilience. Annu. Rev. Psychol..

[11] Schetter C.D., Dolbier C. (2011). Resilience in the Context of Chronic Stress and Health in Adults. Soc. Pers. Psychol. Compass.

[12] Zhang D.J., Feng Z.Z., Guo C., Chen X. (2000). Problems on research of children’s psychological suzhi. J. Southwest Univ. (Soc. Sci. Ed.).

[13] Zhang D.J., Zhang J. (2018). Revision of college students’ Psychological Suzhi Questionnaire (simplified version) and Its Reliability and Validity Test. J. Southwest Univ. (Soc. Sci. Ed.).

[14] Furlong M.J., Gilman R., Huebner E.S. (2014). Handbook of Positive Psychology in Schools.

[15] Zhang D.J., Wang X.Q. (2012). An analysis of the relationship between mental health and psychological suzhi: From the perspective of connotation and structure. J. Southwest Univ. (Soc. Sci. Ed.).

[16] Wu L.L., Zhang D.J., Cheng G., Hu T.Q. (2018). Bullying and Social Anxiety in Chinese Children: Moderating Roles of Trait Resilience and Psychological Suzhi. Child Abus. Negl..

[17] Liu G.Z., Pan Y.G., Li B.B., Hou X.L., Zhang D.J. (2019). The protective effect of psychological suzhi on the relationship between school climate and alcohol use among Chinese adolescents. Psychol. Res. Behav. Manag..

[18] Wen Z.L., Chang L., Hau K.T. (2006). Mediated Moderator and Moderated Mediator. Acta Psychol. Sin..

[19] Huston A.C., Bentley A.C. (2010). Human Development in Societal Context. Annu. Rev. Psychol..

[20] Rosenberg M. (1965). Society and the Adolescent Self-Image.

[21] Ryan R.M., Deci E.L. (2000). Self-determination theory and the facilitation of intrinsic motivation, social development, and well-being. Am. Psychol..

[22] Jiang S. (2020). Psychological well-being and distress in adolescents: An investigation into associations with poverty, peer victimization, and self-esteem. Child Youth Serv. Rev..

[23] Lin C.C. (2015). Gratitude and depression in young adults: The mediating role of self-esteem and well-being. Personal. Individ. Differ..

[24] Xiang Z.L., Tan S., Kang Q., Zhang B.S., Zhu L. (2019). Longitudinal Effects of Examination Stress on Psychological Well-Being and a Possible Mediating Role of Self-Esteem in Chinese High School Students. J. Happiness Stud..

[25] Cong X.B., Tian L.M., Zhang X.K. (2005). Self-esteem: The Core of Mental Health. J. Northeast Norm. Univ..

[26] Yang L.Z., Zhang L.H. (2003). On psychological significance of self-esteem. Psychol. Explor..

[27] Fernandez-Garcia M.P., Vallejo-Seco G., Livacic-Rojas P., Tuero-Herrero E. (2018). The (Ir)Responsibility of (Under)Estimating Missing Data. Front. Psychol..

[28] Li X.B., Wang Z.M., Hou Y.Z., Wang Y., Liu J.T., Wang C.Y. (2014). Effects of childhood trauma on personality in a sample of Chinese adolescents. Child Abus. Negl..

[29] Wu Q.L., Chi P.L., Lin X.Y., Du H.F. (2018). Child maltreatment and adult depressive symptoms: Roles of self-compassion and gratitude. Child Abus. Negl..

[30] Yu G.L., Li S., Zhao F.Q. (2020). Childhood maltreatment and prosocial behavior among Chinese adolescents: Roles of empathy and gratitude. Child Abus. Negl..

[31] Fu W.Q., Yao S.Q., Yu H.H., Zhao X.F., Li R., Li Y., Zhang Y.Q. (2005). Initial reliability and validity of Childhood Trauma Questionnaire (CTQ-SF) applied in Chinese college students. J. Clin. Psychol. Med. Settings.

[32] Curran P.J., West S.G., Finch J.F. (1996). The robustness of test statistics to nonnormality and specification error in confirmatory factor analysis. Psychol. Methods.

[33] Preacher K.J., Hayes A.F. (2008). Asymptotic and resampling strategies for assessing and comparing indirect effects in multiple mediator models. Behav. Res. Methods.

[34] Hayes A.F. (2013). Introduction to Mediation, Moderation, and Conditional Process Analysis: A Regression-Based Approach.

[35] Frazier P.A., Tix A.P., Barron K.E. (2004). Testing Moderator and Mediator Effects in Counseling Psychology Research. J. Couns. Psychol..

[36] Podsakoff P.M., MacKenzie S.B., Lee J.Y., Podsakoff N.P. (2003). Common method biases in behavioral research: A critical review of the literature and recommended remedies. J. Appl. Psychol..

[37] Liu G.Z., Fang L.Y., Pan Y.G., Zhang D.J. (2019). Media multitasking and adolescents’ sleep quality: The role of emotional behavioral problems and psychological suzhi. Child Youth Serv. Rev..

[38] Carver C.S., Connor-Smith J. (2010). Personality and Coping. Annu. Rev. Psychol..

[39] Dong Z.S., Zhang D.J. (2015). The Relationship between psychological Suzhi, emotion regulation strategies and life satisfaction among middle school students. J. Southwest Univ. (Soc. Sci. Ed.).

[40] Liu G.Z., Zhang D.J., Pan Y.G., Chen W.F., Ma Y.X. (2016). The relationship between middle school students’ psychological suzhi and peer relationship: The mediating role of self-esteem. Psychol. Sci..

[41] Pan Y.G., Hu Y., Zhang D.J., Ran G.M., Li B.B., Liu C.X., Liu G.Z., Luo S.L., Chen W.F. (2017). Parental and peer attachment and adolescents’ behaviors: The mediating role of psychological suzhi in a longitudinal study. Child Youth Serv. Rev..

[42] Zhang D.J., Wang J.L., Yu L. (2011). Methods and Implementary Strategies on Cultivating Students’ Psychological Suzhi.

[43] Bajaj B., Gupta R., Pande N. (2016). Self-esteem mediates the relationship between mindfulness and well-being. Pers. Individ. Differ..

[44] Randal C., Pratt D., Bucci S. (2015). Mindfulness and Self-esteem: A Systematic Review. Mindfulness.

[45] Pepping C.A., O’Donovan A., Davis P.J. (2013). The positive effects of mindfulness on self-esteem. J. Posit. Psychol..

[46] Menéndez A.G., García P.F., Viejo I.T. (2010). Aceptación del dolor crónico en pacientes con bromialgia: Adaptación del Chronic Pain Acceptance Questionnaire (CPAQ) a una muestra española. Psicothema.

